# Elevated Matrix Metalloproteinase Levels in Bronchi Infected with Periodontopathogenic Bacteria

**DOI:** 10.1371/journal.pone.0144461

**Published:** 2015-12-14

**Authors:** Luca Bernasconi, Liza L. Ramenzoni, Ahmed Al-Majid, Gabrielo M. Tini, Sereina M. Graber, Patrick R. Schmidlin, Sarosh Irani

**Affiliations:** 1 Centre for Laboratory Medicine, Cantonal Hospital Aarau, Tellstrasse, CH-5001 Aarau, Switzerland; 2 Clinic of Preventive Dentistry, Periodontology, and Cariology, Center for Dental Medicine, University of Zurich, Plattenstrasse 11, CH-8032 Zurich, Switzerland; 3 Clinic of Pulmonary and Sleep Medicine, Cantonal Hospital Aarau, Tellstrasse, CH-5001 Aarau, Switzerland; 4 Anthropological Institute and Museum, University of Zürich-Irchel, Winterthurerstrasse 190, CH-8057 Zürich, Switzerland; Stellenbosch University Faculty of Medicine and Health Sciences, SOUTH AFRICA

## Abstract

**Objectives:**

To determine whether bronchial colonisations/infections with periodontopathogenic bacteria are associated with elevated inflammatory markers such as MMPs, interleukins and Tumor necrosis factor alpha in the bronchial fluid.

**Methods:**

Periodontal status was assessed in consecutive outpatients planned for elective bronchoscopies, and PCR for periodontopathogenic bacteria was performed from a protected specimen brush sample taken from the bronchial mucosa. Additionally, MMPs, interleukins and Tumor necrosis factor alpha were measured in the bronchial fluid.

**Results:**

Out of the four species assessed, one species was found in 13 of 91 (14%) patients, and two in 12 (13%), three in 13 (14%) and all four in 1 (1%) patient, respectively. In multiple linear regression models the presence of Treponema denticola showed a consistent pattern of positive effects in bronchial fluid (Bonferroni adjusted p-values) on the levels of MMP9 (p adj.: 0.028) and MMP12 (p adj.: 0.029). Active smoking was independently associated with increased levels of aMMP8 (p adj.: 0.005) and MMP9 (p adj.: 0.009). Levels of IL-1 ß, IL-8 and Tumor necrosis factor alpha measured in the bronchial fluid were not affected by the presence of periodontopathogenic bacteria.

**Conclusions:**

Bronchial colonisation/infection with Treponema denticola and smoking are independently associated with elevated MMPs (MMP9/MMP12 and MMP8/MMP9, respectively) in the bronchial fluid.

## Introduction

Periodontitis is a destructive inflammatory disease of the tooth-supportive tissues, including alveolar bone and gingiva. Bone resorption and tissue destruction are thought to be the result of the imbalance between bacterial colonisation and pathogenic inflammatory host response [1]. *Aggregatibacter actinomycetemcomitans* (Aa), *Porphyromonas gingivalis* (Pg), *Tannerella forsythia* (Tf) and *Treponema denticola* (Td) are considered to be the key pathogenic marker species [2] and are classified together as the red complex in Socransky’s subgingival cluster model [3].

Matrix metalloproteinases (MMPs) represent a group of structurally similar enzymes that play a central role in inflammation related tissue degradation. In particular MMP8 and MMP9 in gingival fluid have been shown to be associated with periodontitis [4–6]. Furthermore, recent studies showed elevated MMPs in bronchoalveolar lavage fluid in bacterial pneumonia [7] and experimental lung injury [8,9]. MMPs and local levels of inflammatory mediators such as Interleukin 1beta (IL1-ß), Interleukin 8 (IL-8) and Tumor necrosis factor alpha (TNF-α) were also found to be elevated in periodontitis [10,11] as well as in infectious lung diseases [12].

The lower respiratory tract (LRT) is no longer believed to be sterile, mainly as a result of culture independent techniques, and its’ microbiome became a focus in healthy [13] and diseased lung research [14–16]. In spite of the close anatomical proximity of the oral and bronchial compartments, to date only few studies have addressed the colonisation of the lung with periodontal pathogens. In a recent pilot study this research group was able to find key pathogenic marker species in the lungs of lung transplant recipients despite the frequent use of antibiotics in this population [17]. Recently, we have also shown in a larger population that periodontal key pathogenic marker species can be frequently detected in the bronchial compartment [18]. It appears, therefore, that the oral and the bronchial compartment share some colonisation patterns, particularly with regard to specific pathogenic key marker species. The preliminary and semi-quantitative data found in this study also demonstrated that bronchial colonisation/infection with these species seemed to be associated with increased aMMP8 levels (semi-quantitatively assessed) in the bronchial fluid. Furthermore, periodontitis appeared to represent an independent risk factor for bronchial colonisation/infection with the pathogenic marker species of the red complex mentioned earlier [18].

Therefore the aim of the current study was to test the hypothesis that comparably to gingival structures of the oral cavity, colonisation/infection in the lung with key pathogenic marker species is also associated with increased levels of MMPs and other inflammatory markers, i.e. cytokines, such as IL1-ß, IL-8 and TNF-α. In addition, assessment was made as to whether periodontitis was associated with increased levels of the aforementioned markers.

## Materials and Methods

Outpatients between 18 and 80 years of age, scheduled for elective bronchoscopy in Cantonal Hospital, Aarau, were included in the current prospective study once their written informed consent was obtained. Exclusion criteria included the use of systemic or local antibiotics during the preceding six weeks, less than four teeth or severe coagulopathy. In all patients, the entire range of clinical information was available, as well as a body plethysmography (MasterScreen, Jaeger, Hoechberg, Germany), which had been performed under stable clinical conditions within one month prior to bronchoscopy. The study protocol was approved by the Ethics Committee of the Canton Aargau, Switzerland (EK 2012/018).

The periodontal status assessment, the bronchoscopy procedure and the bronchial sample collection have been described in detail previously [18]. In brief, a periodontal screening index (PSI) was determined with the aid of a pressure-sensitive probe (Hawe Click Probe, Kerr Hawe, Bioggio, Switzerland) [19]. Scores 0 to 4 were defined by assessment of probing pocket depth and bleeding, (where scores 0, 1 and 2 infer no periodontits, and scores 3 and 4 infer periodontitis) [20]. Trans-nasal bronchoscopy was carried out under sedation with propofol (Disoprivan, Astra Zeneca, Zug, Switzerland) and local anesthesia of the vocal cords with 10 ml of 1% lidocaine. The endoscope was placed directly into the middle lobe bronchus (or upper lobe bronchus), without suction, and with the aid of a wax-plug protected specimen brush (PSB) (Vygon, Niederwangen, Switzerland) the mucosa was sampled using a standardised technique. Furthermore, 8 ml of physiologic saline was instilled into the lingua bronchus (or upper lobe bronchus) and subsequently re-aspirated. The aspirate was frozen at -80°C and referred for the measurement of inflammatory markers. Finally, another 8ml of physiologic saline was instilled and the aspirate was then referred for conventional microbiology. Immediately after the procedure the PSB was placed in a transport vial and the bronchial fluid was frozen at -80°C. In five patients sham PSB samples were taken in addition to the described procedure (i.e. a complete procedure with PSB sampling but without mucosa contact of the brush).

Using a commercially available PCR test (micro-IDent test, Hain Lifescience, Nehren, Germany) [21] four key pathogenic marker species (Aa, Pg, Tf and Td) were determined in the PSB sample (for details see [18]). In contrast to the original test, which applies semi-quantitative results, increased cycles numbers of were used. Therefore, the results can be considered as qualitative rather than quantitative.

Measurement of MMP-2 and MMP-12 was employed as being representative of biomarkers of tissue damage, which have been investigated in lung diseases, to a certain degree [8,22,23]. MMP9 and MMP8 were chosen, since they are intensively investigated in periodontal research, in particular MMP8 in its activated form. MMP-2, MMP-9 and MMP-12 levels were measured using a multiplexed particle-based flow cytometric cytokine assay (Luminex Performance Assay Human MMP, R&D Systems, USA) [24]. MMP2 and MMP9 (both detect pro- and mature MMP forms; #LMP902C and #LMP911B); MMP12 (detect pro-, mature, and TIMP1 complexed MMP12; #LMP919B). The procedures closely followed the manufacturer’s instructions. The analysis was conducted using a conventional flow cytometer (Guava EasyCyte Plus, Millipore, Zug, Switzerland).

The serum was assayed according to the manufacturer’s instructions using a commercially available quantitative enzyme-linked immunosorbent assay (ELISA) for aMMP8 (Dentognostics, Matrix Biotech, Jena, Germany) and Interleukin 1beta (IL1-ß), Interleukin 8 (IL-8) and Tumor necrosis factor alpha (TNF-α) (Life technologies, Termo FischerScientific, Waltham, MA, USA). Assays were performed in duplicate, and the optical density at 450nm was determined, with background correction 570nm, using a microplate reader (Labsystems iEMS Reader MF). The reading for each standard and sample was averaged and the zero standard average was subtracted. The lower limits of detection were as follows: aMMP8, 6pg/ml; IL1-ß, 2pg/ml; IL-8, 1pg/ml; TNF-α, 3pg/ml.

Statistica software (release 10, StatSoft Inc., Tulsa, USA) and R programming language (R Core Team (2012). R: A language and environment for statistical computing. R Foundation for Statistical Computing, Vienna, Austria. ISBN 3-900051-07-0) were used for statistical analyses. Categorical data are presented as absolute numbers (percentages), continuous data as median (interquartile range (IQR)). The non-parametric Mann Whitney U test and chi-square test were used to compare groups, where appropriate. In accordance with the research hypothesis, multiple linear regression models were used to test the effects of the presence of the three key pathogenic marker species (Pg, Tf and Td) assessed in the PSB sample (absence/presence: see methods), periodontitis (absence/presence of PSI > 2: see methods) and active smoking (absence/presence) on the level of the MMPs determined in the bronchial fluid. As a consequence of too few events, Aa was not included in the model. One model was used for each of the four MMPs (MMP2, aMMP8, MMP9 and MMP12) and p-values were correspondingly adjusted for multiple testing (Bonferroni). Continuous response variables were either natural Log or box-cox (power transformation y^λ^) transformed in order to reach appropriately distributed residuals. Active smoking was included in the model since elevated MMPs have been shown in the periodontal tissue of smokers [25].

## Results

**Fig 1:**
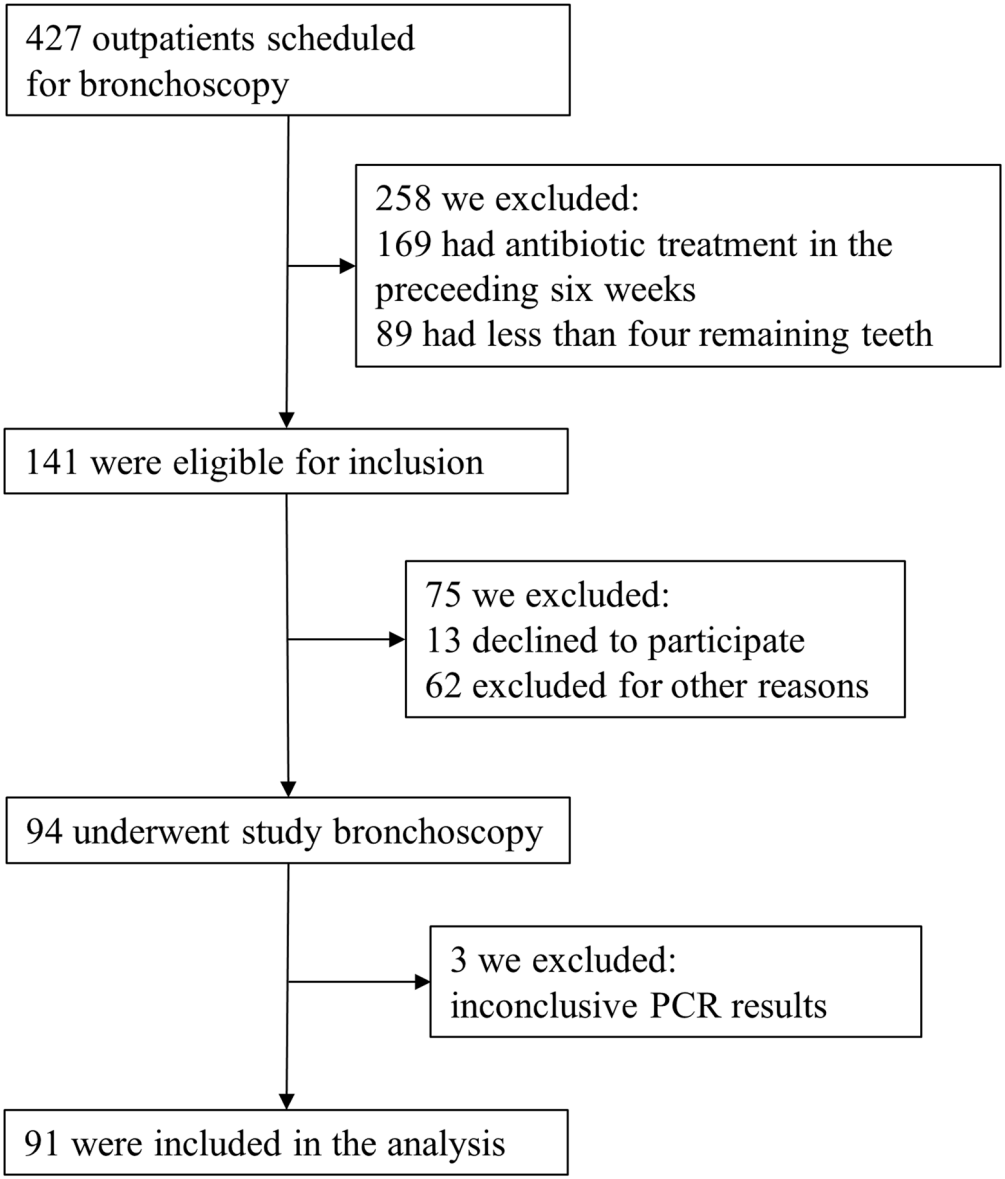
Enrolment of study participants.

Fig 1 shows the enrolment of the participant patients. All bronchoscopies were performed without complication. The demographic and clinical data of the study population at baseline are summarised and compared separately for patients with and without Td in Table 1 (the grouping was chosen due to the impact of Td on MMPs mentioned later in the manuscript). In the 91 patients, the PCR of the PSB lung sample for Aa, Pg, Tf and Td was positive in 4 (4%), 28 (31%), 27 (30%) and 21 (23%), respectively. In 13 (14%) patients one of the four species was found, in 12 (13%) two species, in 13 (14%) three species and in 1 (1%) four species. PCR of all five PSB sham samples was negative for all of the four key pathogenic marker species.

**Table 1:** Demographic and clinical data of the study population.

	Td bronchial—(n = 70)	Td bronchial + (n = 21)	
Characteristic			p
Age, years	60 (48 to 71)	59 (48 to 64)	0.581
Gender, female	23 (33)	6 (29)	0.711
Smoking status			
Current smoker	25 (36)	4 (19)	0.151
Former smoker	22 (31)	6 (29)	0.803
Never smoker	23 (33)	11 (52)	0.105
COPD	15 (21)	6 (29)	0.495
Gold I	3 (4)	4 (19)	
Gold II	9 (13)	1 (5)	
Gold III	2 (3)	1 (5)	
Gold IV	1 (1)	0 (0)	
Lung cancer, yes	23 (33)	5 (24)	0.431
Periodontal status			
PSI 0	23 (33)	4 (19)	0.224
PSI 1–2	36 (51)	8 (38)	0.283
PSI 3–4	11 (16)	9 (43)	0.008

Td:Treponema denticola, COPD:chronic obstructive pulmonary disease (Classification according to the degree of airflow limitation I to IV: forced volumen in one second ≥80% predicted, ≥50–80% predicted, ≥30–50% predicted and <30% predicted, respectively), PSI = periodontal screening index (Classification according to the probing pocket depth and bleeding 0 to 4: scores 0, 1 and 2: no periodontitis, scores 3 and 4: periodontitis)

Data presented as median (IQR) or number (%), Mann-Whitney U and Chi-square test were used where appropriate

In 32 participants (35%), the following potentially pathogenic bacteria were found in the bronchial fluid conventional cultures: *Haemophilus influenza* 12 (13%), *Haemophilus parainfluenzae* 14 (15%), *Klebsiella pneumonia* 2 (2%), *Escherichia coli* 1 (1%), *Staphylococcus aureus* 3 (3%), *Pseudomonas aeruginosa* 1 (1%), *Streptococcus pneumonia* 1 (1%) and *Serratia marcescens* 1 (1%). These microorganisms were not considered clinically relevant in any of the patients.

The results of the bronchial fluid immunoassays for MMPs and interleukins in the overall population investigated were as follows (Median, (IQR)): MMP2 639 (309–2215) pg/ml, aMMP8 215 (91–492) ng/ml, MMP9 105.6 (52–265.8) ng/ml, MMP12 5 (5–20.7) pg/ml, IL1ß 15.3 (13.5–16.7) pg/ml, IL8 10.4 (8.3–11.8) pg/ml and TNF-α 16.5 (13.8–20.5) pg/ml.

**Fig 2:**
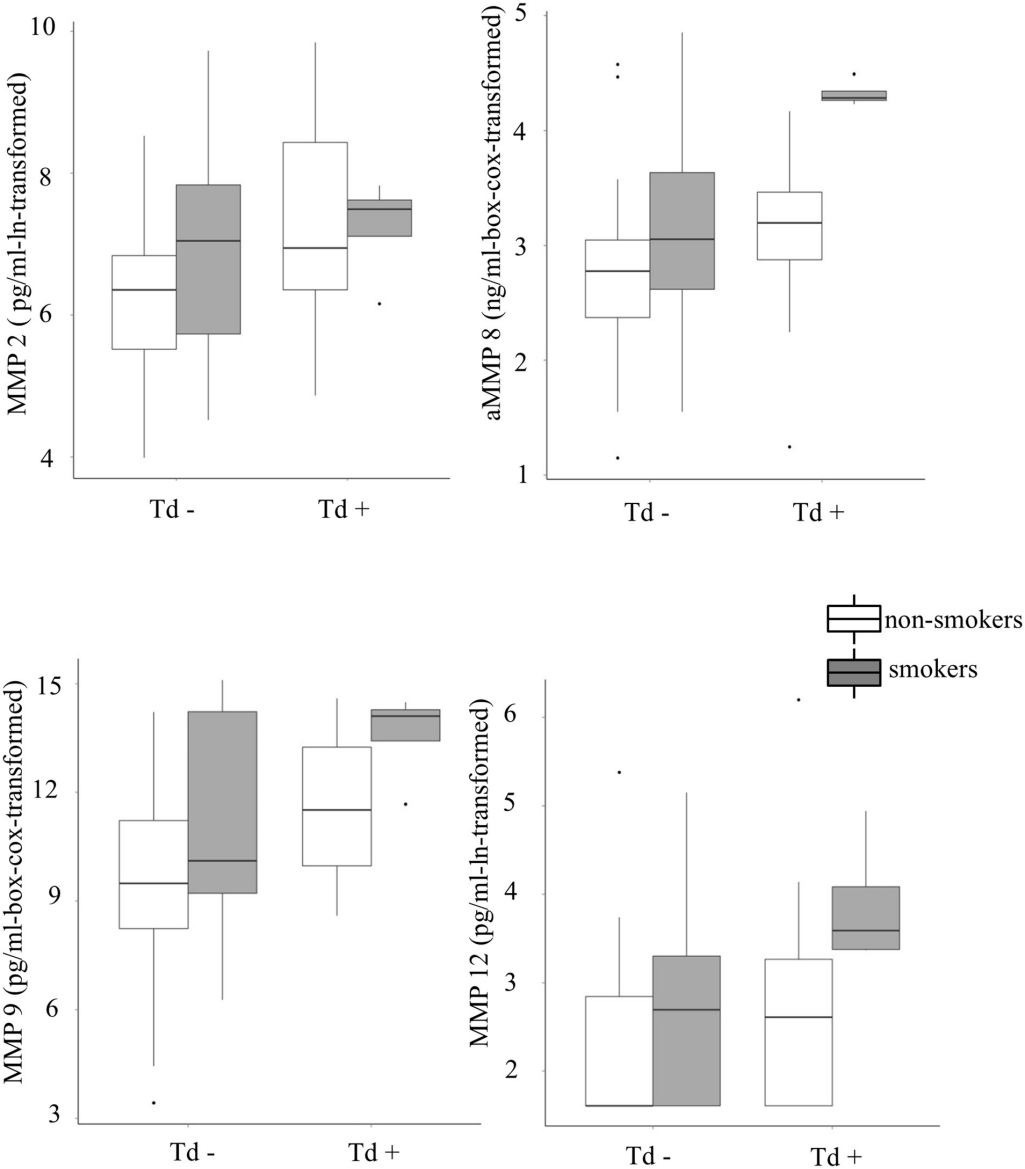
Levels of matrix metalloproteinases in the bronchial fluid of patients, with and without bronchial colonisation with Treponema denticola (Td), shown separately for active smokers (dark grey) and non-smokers (transparent). The y-axes are either ln- or box-cox-transformed, corresponding to the transformations used in the multiple regression models. The dots indicate outliers.

**Fig 3:**
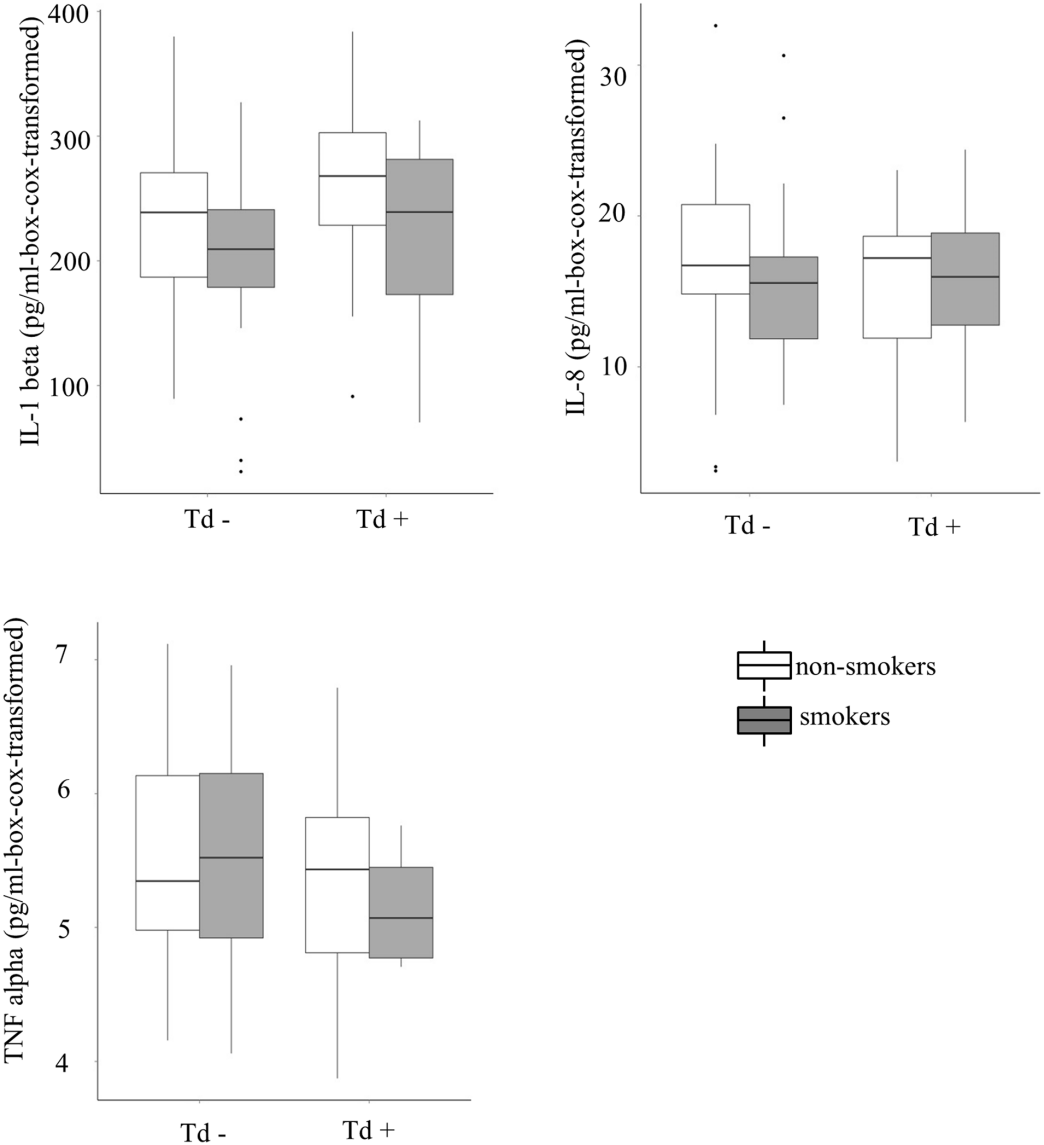
Cytokine levels in the bronchial fluid of patients with and without bronchial colonisation with Treponema denticola (Td), shown separately for active smokers (dark grey) and non-smokers (transparent). The y-axes are box-cox-transformed, corresponding to the transformations used in the multiple regression models. The dots indicate outliers.

The results of the multiple linear regression models testing the effects of the three key pathogenic marker species (Pg, Tf and Td), the presence or absence of periodontitis and the presence or absence of active smoking on the four MMPs are given in Table 2. Models including Aa lead to absolutely equivalent results, however, due to infrequent events, it was decided to exclude it from the final models. Among the three pathogenic markers, Td shows a consistent positive effect pattern regarding the levels of the four MMPs (Fig 2). Interaction terms were non-significant and therefore were excluded from the models. The same models were applied to test the effects on IL1-ß, IL-8 and TNF-α, however, a statistically significant effect of active smoking was shown only in case of IL1-ß (p = 0.009, results not numerically shown, see Fig 3). Further, we included COPD or the presence of potentially pathogenic bacteria found in the conventional culture, as a covariate in these models. However, neither affected MMPs, cytokines or any of the results mentioned above (data not shown).

**Table 2:** Multiple linear regression models with the response variable given by the level of matrix metalloproteinases in bronchial fluid. Given are the coefficient of multiple determination (R2), estimates, standard error, the corresponding p-values and the corresponding p-values adjusted for multiple testing (Bonferroni). Signicant p-values are indicated in bold font (i.e. < 0.05).

Response variable	R^2^	predictors	Estimate	Std. Error	p	adj. P
MMP2	0.114	Pg+ bronchial	-0.603	0.346	0.085	0.340
		Tf+ bronchial	0.122	0.364	0.737	1.000
		Td+ bronchial	0.988	0.408	0.018	0.071
		Periodontits (PSI>2)	0.119	0.344	0.730	1.000
		Active smoking	0.485	0.293	0.102	0.407
aMMP8	0.180	Pg+ bronchial	-0.089	0.203	0.664	1.000
		Tf+ bronchial	-0.011	0.213	0.957	1.000
		Td+ bronchial	0.593	0.240	0.016	0.062
		Periodontits (PSI>2)	0.066	0.203	0.746	1.000
		Active smoking	0.573	0.171	0.001	0.005
MMP9	0.203	Pg+ bronchial	-0.009	0.644	0.989	1.000
		Tf+ bronchial	0.090	0.677	0.895	1.000
		Td+ bronchial	2.103	0.760	0.007	0.028
		Periodontits (PSI>2)	0.300	0.640	0.640	1.000
		Active smoking	1.711	0.546	0.002	0.009
MMP12	0.149	Pg+ bronchial	-0.028	0.273	0.919	1.000
		Tf+ bronchial	-0.285	0.287	0.323	1.000
		Td+ bronchial	0.886	0.322	0.007	0.029
		Periodontits (PSI>2)	0.130	0.271	0.632	1.000
		Active smoking	0.569	0.231	0.016	0.063

MMP:matrix metalloproteinase, Pg: Porphyromonas gingivalis, Tf: Tannerella forsythia, Td:Treponema denticola, coded yes/no (see methods), PSI = periodontal screening index (Classification according to the probing pocket depth and bleeding 0 to 4: scores 0, 1 and 2: no periodontitis, scores 3 and 4: periodontitis).

## Discussion

Lung microbiome has gained interest in the literature, in particularly, it has been recognised that even in asymptomatic patients, lung microbiome does not merely reflect the bacterial composition of the upper airways [26]. Recently we have shown in a relatively large population that periodontal pathogenic species can be frequently detected in bronchi [18]. Therefore, the current observational study investigated the hypothesis that a bronchial colonisation with the four key pathogenic marker species Aa, Pg, Tf and Td is associated with similar local inflammatory reactions as observed in affected periodontal sites.

MMPs seem to represent important peptides and markers in both, the pathogenesis of lung diseases [7,9,27,28] and periodontitis [5,25,29]. Therefore, in the current study MMP2, aMMP8, MMP9 and MMP12 were measured in the bronchial fluid of 91 patients undergoing elective diagnostic bronchoscopy and these findings correlated with the presence of periopathogenic marker species. Several of our observations were remarkable as expected.

Firstly, we found that the presence of Td in the bronchial compartment was associated with increased levels of MMPs, particularly aMMP8 and MMP9. This is in agreement with recent studies [4,30,31], which measured increased MMPs in the gingival crevicular fluid of patients with infected periodontal tissues. In addition to the four key pathogenic marker species Aa, Pg, Tf and Td Yakob et al. [4] also performed PCR in gingival fluid for *Prevotella intermedia*. The authors found that increased levels of MMP8 in the gingival crevicular fluid were associated with the presence of Tf and Td whereas increased MMP9 was exclusively associated with Td. This was corroborated by the present study, which also found significantly increased levels of MMP9 in the bronchial fluids, which were strongly associated with the presence of Td. In contrast, for Tf no significant association was found. This may be partly the consequence of the fact, that the activated form of MMP8 was measured in this study. In the case of periodontitis the interaction of Td and oral host proteins has been studied many times, and extensively reviewed [32]. It seems that bronchial colonisation/infection with Td is associated with local pro-inflammatory processes in comparable ways. Although increased levels of MMP8 and MMP9 of bronchoalveolar lavage fluid in earlier studies have been associated with severe infectious conditions such as ventilator associated pneumonias [7,33],the exact role of MMPs in inflammatory diseases of the lung is not yet understood. Data concerning levels of MMPs and development or progression of COPD for example, are conflicting [27,34]. Cell surface lipopolysaccharides of Td that bind to extracellular matrix components of the periodontal tissue seem to induce pro-inflammatory pathways, which eventually contribute to the development of periodontitis [35]. In the current study evidence was found that Td is associated with elevated MMPs in the bronchial compartment. However, whether comparable inflammatory mechanisms underlie in the periodont and in the bronchial compartment, respectively, needs to be investigated in future studies. So far, beside… (hier muss noch MMP2, MMP12 in der Lunge erörter warden; MMP9 und 8 ist oben erwähnt).

Secondly, smokers have shown increased levels of aMMP8 and MMP9 in the bronchial fluid independently of the presence of Td. This finding shows similarities with recent observations reported by Victor and co-workers [25]. In the latter study, MMP8 and MMP9 were determined in the gingival crevicular fluid of smokers and non-smokers suffering from chronic periodontitis. Smoking and periodontitis were both associated with increased levels of MMP8 and MMP9 in gingival crevicular fluid in the sense of additional factors. Increased levels of MMP8 and MMP9 in the bronchoalveolar lavage fluid of smokers [34] and induced sputum of smokers [23], respectively, have been reported earlier. However, to our knowledge, the current study was the first to demonstrate that smoking and the presence of Td in the bronchial compartment are independently associated with increased levels of aMMP8 and MMP9 in an additional sense.

Interestingly, in the case of the cytokines IL1- ß, TNF-α and IL-8, consistent patterns could not be found. Noh et al. [11] found increased IL8 and TNF alpha levels in gingival tissue of patients suffering from periodontitis. Although, IL1-ß,and IL-8 have been found to be elevated in the bronchoalveolar lavage fluid of patients suffering from ventilator associated pneumonias [12], it appears that in contrast to MMPs the levels of these cytokines in the bronchial fluid are not consistently altered in the case of smoking or colonisation/infection with periopathogens. This may be the consequence of a more discrete inflammation in the latter cases. Furthermore, it was likely that cytokines secreted in focal areas of inflammation in the lungs were degraded locally or removed in the circulation before they diffused into the bronchial fluid.

Somewhat surprisingly and apparently in contrast to other authors [23,27,34] we could not correlate the levels of MMPs measured in bronchial fluid with the presence or absence of COPD. However, taking a closer look in one study [27], no information was provided regarding the smoking status of the study participants. In another study [23], which investigated the levels of MMP8, MMP9 and MMP12 in the induced sputum, only MMP8 was higher in symptomatic smokers with COPD Stage GOLD 0 compared to non-symptomatic smokers. In this study no information is available concerning activated MMP8. Finally, D’Armiento et al. [34] found significantly elevated MMP9 in bronchoalveolar lavage fluid in both smokers and former smokers with emphysema compared to non-smoking controls. However, MMP9 neither correlated with the severity of COPD nor with the degree of disease progression over an 18- month period. Although the role of MMPs and its’ polymorphisms for the development of COPD are discussed extensively in the literature (summarised in [28]), the exact part of MMPs in COPD pathogenesis is not well characterised as yet.

In an earlier study [18] this research group demonstrated that periodontitis appeared to be a risk factor for bronchial colonisation/infection with periopathogens. Therefore, the group expected its current analysis to show that patients suffering from periodontitis might also be more vulnerable to pro-inflammatory consequences of colonisation/infection with periopathogens in the bronchial compartment. However, no evidence was found to support this hypothesis. This may be consequent on having information about the earlier treatment of periodontitis in the participant population, and potentially this interacted with the presence or absence of periodontitis at the time of the study investigation.

The current study is subject to some limitations. Although, to the best of our knowledge so far no data exist concerning inflammatory consequences of bronchial colonisation/infection with periopathogens based on our data we are not able to characterise the clinical implications of these findings in our population. This outcome may be the consequence of the relatively small number of patients and their heterogeneity. In addition, the relatively limited scientific data available concerning the role of MMPs in lung disease and the cross sectional design of the current study hinder the clinical interpretation of our results Due to the relatively small number of patients the impact of potential additional factors on the levels of inflammatory markers could not be analysed in the regression models employed. Another shortcoming is the fact, that tissue inhibitors of metalloproteases were not measured in the current study. Furthermore, although evidence was found for local inflammatory reactions in the case of Td, these researchers could not differentiate between detection, colonisation and infection.

In summary, this cross sectional study has shown that smoking and bronchial colonisation/infection with Td are independently associated with elevated MMPs (aMMP8/MMP9 and MMP9/MMP12, respectively) in the bronchial fluid in a relatively large population.
